# Stiff-person syndrome mimic secondary to hypopituitarism: a case report and literature review

**DOI:** 10.3389/fendo.2025.1664695

**Published:** 2025-10-10

**Authors:** Menghan Zheng, Bo Cui, Junqi Liu, Huanli Duan, Wei Wang, Haifeng Li, Lu Lu

**Affiliations:** ^1^ Innovation Center for Neurological Disorders and Department of Neurology, Xuanwu Hospital, Capital Medical University, National Clinical Research Center for Geriatric Diseases, Beijing, China; ^2^ Department of Neurology, Xuanwu Hospital, Capital Medical University, Beijing, China; ^3^ Department of Otorhinolaryngology Head and Neck Surgery, Xuanwu Hospital, Capital Medical University, Beijing, China; ^4^ Department of Pathology, Xuanwu Hospital, Capital Medical University, Beijing, China

**Keywords:** stiff-person syndrome, hypopituitarism, hypoadrenalism, hyponatremia, hormone replacement therapy

## Abstract

**Introduction:**

Flexion contracture has been reported to be associated with hypopituitarism and hypoadrenalism. We reported a case of a patient who presented with stiff-person syndrome (SPS) mimic secondary to prolactinoma-associated hypopituitarism.

**Methods:**

A case of SPS mimic secondary to hypopituitarism was reported. Literature review identified six additional reported SPS mimic cases associated with hypopituitarism until July 2025. We summarized the etiologies, clinical features, and therapeutic revelations of these cases.

**Results:**

We described a patient who developed progressive rigidity of the lower limbs and trunk with painful spasms precipitated by sensory stimuli for 6 months, initially suspected as SPS. Investigations indicated hypopituitarism secondary to prolactinoma, and hormone replacement therapy showed a favorable clinical response. The literature review showed six similar cases of SPS mimic secondary to hypopituitarism. The underlying causes were attributed to pituitary mass and Sheehan’s syndrome for male and female patients, respectively. Flexion contracture and painful spasms predominated the typical pictures and responded satisfactorily to glucocorticoid supplementation.

**Conclusions:**

SPS mimic is a rare neurological manifestation secondary to hypopituitarism, typically hypoadrenalism. Our report raises awareness of this potential complication to promote early hormonal evaluation and prompt glucocorticoid replacement therapy. Further studies are warranted to elucidate the mechanism between adrenal deficiency and neurological manifestations.

## 1 Introduction

Stiff-person syndrome (SPS) is a rare neurological disorder regarded as an autoimmune neuronal hyperexcitability disease, generally associated with antibodies against glutamic acid decarboxylase (GAD) and thus classified as a GAD antibody spectrum disorder (1). The classic SPS is characterized by progressive rigidity primarily affecting the lower limbs and trunk muscles, accompanied by painful spasms precipitated by startling stimuli. The typical electromyographic (EMG) pattern shows a continuous firing of motor-unit activity that is hardly suppressed voluntarily (2, 3). Broader subtypes have been recognized beyond the classic form, including paraneoplastic SPS and progressive encephalomyelitis with rigidity and myoclonus, which are associated with antibodies against amphiphysin, gephyrin, or glycine receptors (4). The diagnosis is further complicated in cases of seronegative SPS, as well as in SPS mimics—similar presentations of SPS due to other disease entities (5).

Hypopituitarism is an endocrine disorder characterized by partial or complete deficiency of pituitary hormones. The underlying etiologies are highly heterogeneous, ranging from common sellar masses to rare genetic mutations (6). Clinically, hypopituitarism encompasses various subtypes, namely, hypoadrenalism, hypothyroidism, or hypogonadism. Depending on the severity and extent of the pituitary axes’ dysfunction, the manifestations vary from chronic insidious symptoms to acute life-threatening crises. Given the close interaction between endocrinology and neurology, certain neuromuscular dysfunctions are attributed to hormone disturbances in hypopituitarism that may resemble primary neurological disorders. This overlap often renders challenges in early identification and frequently resulted in underdiagnosis and mismanagement (7).

Here, we report a case of SPS mimic secondary to prolactinoma-induced hypopituitarism, and review similar SPS mimic cases due to hypopituitarism that have been published to date.

## 2 Materials and methods

### 2.1 Case presentation

A 68-year-old man presented with a 6-month history of progressive stiffness that started from the bilateral lower limbs, ascending to the body trunk and waist and leading to a fixed deformity. The stiffness was prone to be superimposed by minor tactile stimuli, and considerable time and effort were required for him to be fully relieved to ambulation. There was no significant medical, family, or psychosocial history with the exception of a previous myocardial infarction. Neurologic examination revealed an alert and fully oriented man with restricted upward gaze. Muscle tone was persistently increased in bilateral lower limbs and was elicitable in the upper limbs and abdominal muscles upon tactile stimuli. Tendon reflexes were generally reduced. Postural abnormalities were marked by flexion contracture and difficulty in initiating gait. The distribution and trigger of rigidity were in concordance with classic SPS, and his vertical oculomotor palsy was initially considered as an unusual abnormality occurring in SPS and GAD antibody spectrum disorder-related cerebellar ataxia.

**Figure 1:**
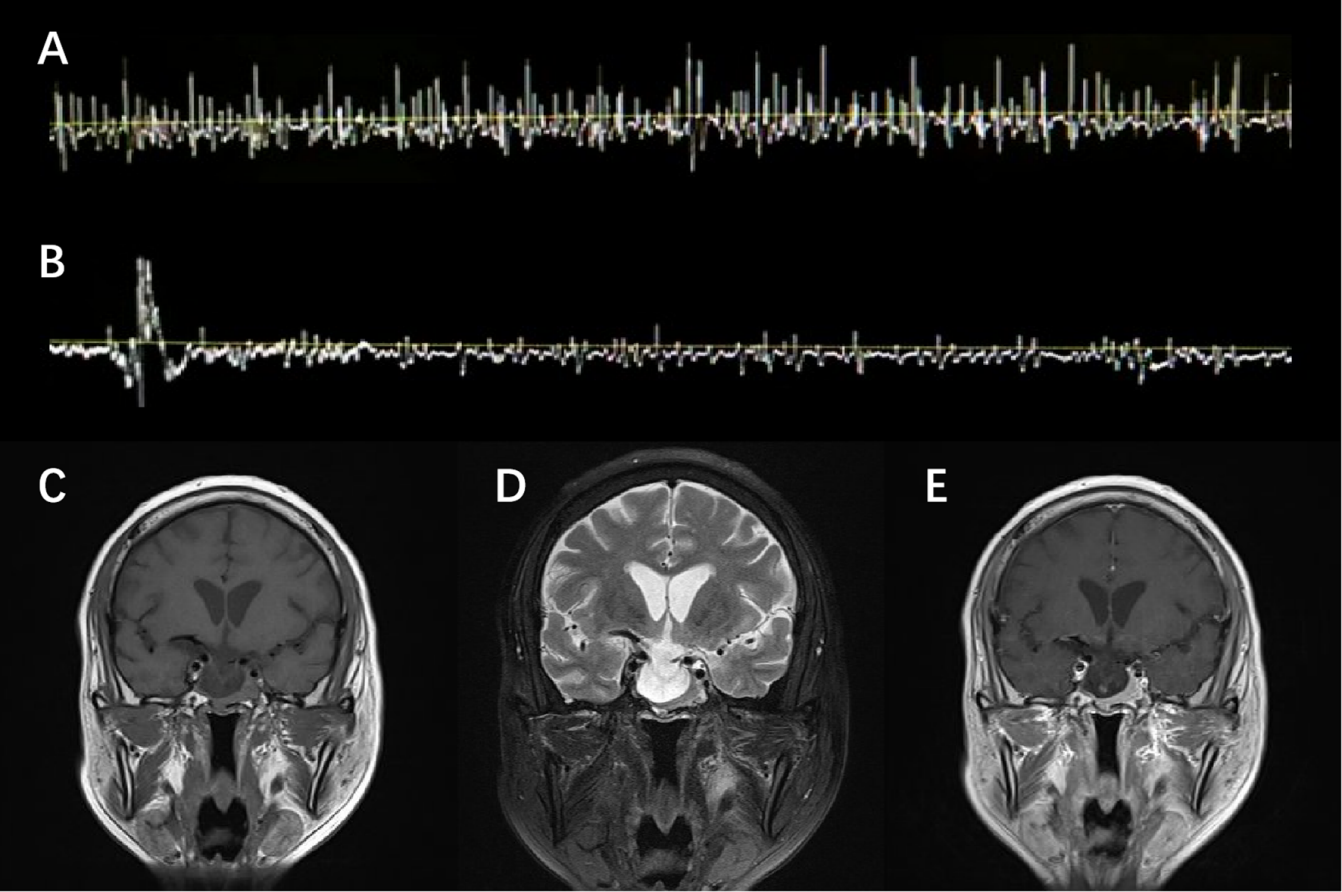
EMG and MRI presentation. EMG observed spontaneous but transient motor-unit potential firing of the right rectus abdominis at the resting state **(A)**, which self-relieved in 4 min without diazepam **(B)**. T1-weighted **(C)**, T2-weighted **(D)**, and Gd-enhanced T1-weighted **(E)** MRI scan of the brain showed a heterogeneously enhanced pituitary and empty sella.

He was admitted with a suspected diagnosis of SPS and underwent relevant examinations. Laboratory tests were largely unremarkable except for inflammatory indicators and thyroid function panels, namely, an elevated erythrocyte sedimentation rate (ESR) of 33 mm/h, a borderline positive result of respiratory syncytial virus (RSV) IgM, and a decreased free thyroxine (FT4) level of 0.39 ng/dL (**Supplementary Table**). A lumbar puncture was performed, revealing cerebrospinal fluid (CSF) cell counts and a protein level of 4×10^6^/L and 68.6 mg/dL, respectively. Despite an increased 24-h intrathecal IgG synthesis rate of 13.55 mg/24 h, the findings of type IV oligoclonal bands and a CSF serum albumin ratio of 9.46×10^−3^ indicated the breakdown of the blood–brain barrier and the absence of intrathecal IgG synthesis. No anti-GAD or anti-amphiphysin antibodies—both known to be pathogenic for SPS—were detected in either serum or CSF. EMG observed paroxysmal motor-unit potential firing of the right rectus abdominis at the resting state, which self-relieved after 4 min without using diazepam (**Figures 1A,B**). Clonazepam 1 mg daily was administered as standard SPS treatment, but no expected improvement in stiffness was observed. These atypical features raised red flags, promoting alternative explanations beside the diagnostic hypothesis of SPS.

Though clonazepam was gradually withdrawn due to the side effects of drowsiness and feebleness, the symptoms persisted and rapidly progressed to somnolence and delirium. The electrolyte result reported a severe hyponatremia with a sodium level of 121 mmol/L. Standard intravenous and oral sodium supplementation was initiated, which proved ineffective in the subsequent 2 days. Further investigations were thus conducted. Brain magnetic resonance imaging (MRI) revealed a homogeneously enhanced pituitary with an empty sella, redirecting an alternative diagnosis to the underlying pituitary pathology (**Figures 1C–E**). A pituitary crisis was suspected based on an abnormal pituitary function panel (**Supplementary Table**), particularly disruption of gonadal function (FSH 0.97 mIU/mL, LH 0.38 mIU/mL, and PRL >204 ng/mL). Given the infection upon admission, the pituitary decompensation was presumably attributed to precipitated inflammatory stress. Meanwhile, the patient’s condition further deteriorated to the extent of developing acute coronary syndrome when the Troponin I level peaked at 4.636 ng/mL and the electrocardiogram showed ST-segment depression in the inferior and extensive anterior leads.

For treatment, acyclovir, ceftriaxone, and intravenous immunoglobulin (10 and 25 g/day for 3 days each) were administered to address potential infection. In addition, intravenous corticosteroid supplement was initiated with hydrocortisone 200 mg/day for 5 days, followed by methylprednisolone 1,000 mg/day for 3 days with a tapering of 500, 240, and 120 mg/day for 1 day each. Conservative strategies, mainly dual antiplatelet and warfarin, were applied for the cardiovascular event. Two days after the addition of hydrocortisone 200 mg/day, there was a dramatic improvement in both consciousness and stiffness, accompanied by normalization of sodium level to 140 mmol/L. Over the following 10 days, the patient’s consciousness gradually returned to normal. Meanwhile, he exhibited significant improvement in stiffness and ambulation without assistance. Neurologic examination revealed an alert man with mild cognitive impairment. Increased muscle tone remained, but tendon reflexes recovered normally in the upper limbs, with a bilateral positive Babinski sign. Plasma sodium level stabilized at 135 mmol/L and Troponin I recovered to 0.009 ng/mL. The patient was subsequently discharged with a prescription of prednisone 60 mg, levothyroxine 25 μg, and bromocriptine 2.5 mg daily.

**Figure 2:**
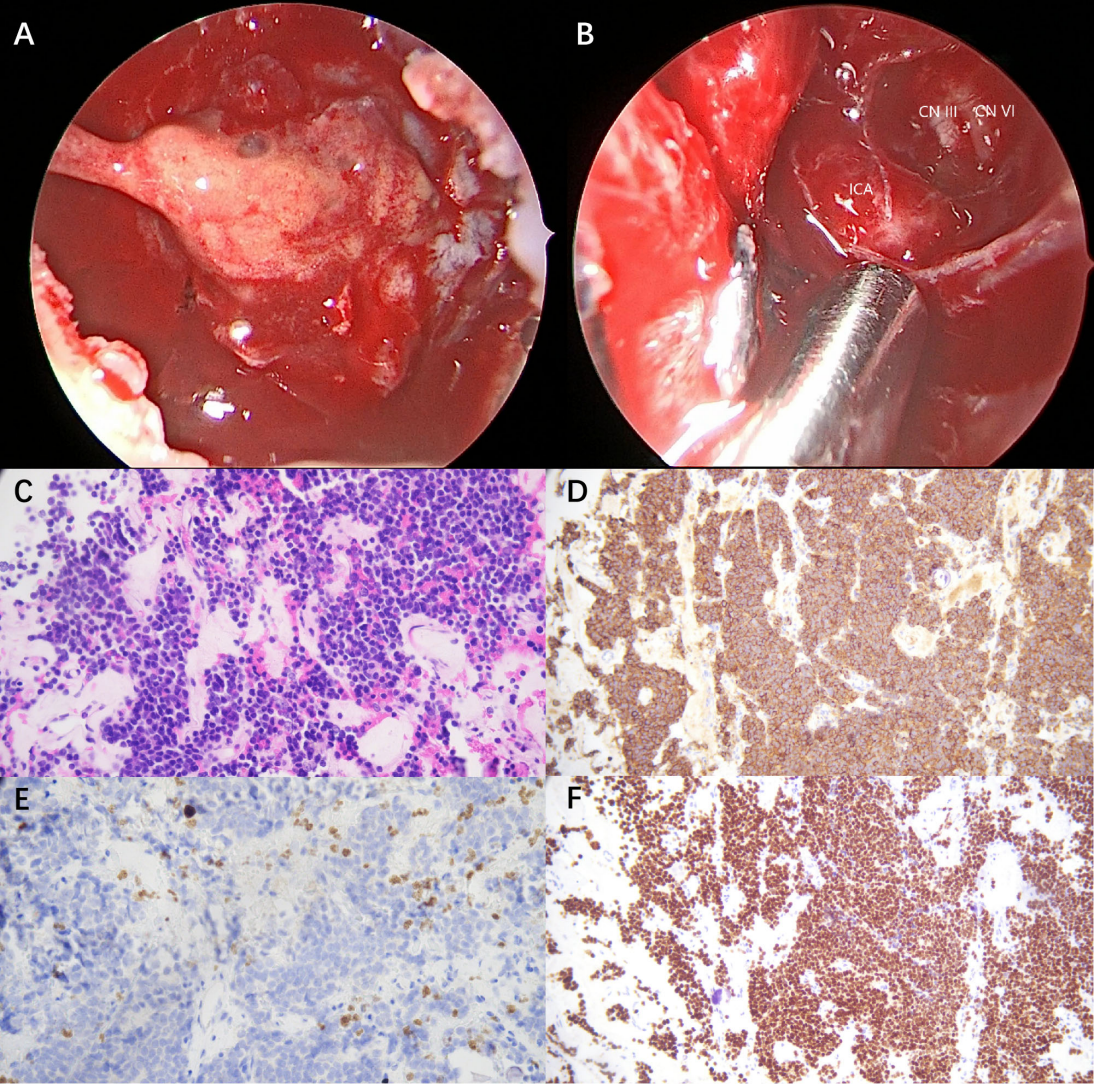
Intra-operative observation and post-operative biopsy (×200). **(A)** A yellow gelatinous tumor invaded into the cavernous sinus, wrapping the internal carotid artery and compressing cranial nerve III and VI. **(B)** Oculomotor nerve (CN III), abductor nerve (CN IV), and internal carotid artery (ICA) were exposed after tumor resection. **(C)** Hematoxylin and eosin (H&E) staining demonstrated uniform tumor cells with round nuclei and eosinophilic cytoplasm. **(D)** Immunohistochemistry for prolactin (PRL) revealed diffuse strong cytoplasmic positivity. **(E)** Ki-67 staining demonstrated a low proliferative index of 2%. **(F)** Pit-1 immunostaining revealed diffuse nuclear positivity.

A pituitary operation was performed 1 month later, which revealed a yellow gelatinous tumor that invaded into the cavernous sinus, wrapping the internal carotid artery and compressing cranial nerve III and VI (**Figures 2A,B**). Post-operational biopsy confirmed the diagnosis of invasive macroprolactinoma (**Figures 2C–F**). PRL returned to physiological ranges 5 days after the total tumor resection. The patient’s clinical manifestations of pyramidal tract signs and contractures, as well as biochemical abnormalities, resolved completely in the 1-year follow-up. Yet, his mild cognitive impairment remained until the end of the follow-up.

### 2.2 Literature review

A systematic literature review was conducted in PubMed and Google Scholar to identify relevant articles reporting SPS mimics associated with hormone disturbances. The search was performed using the search term [(hypopituitarism) OR (hypoadrenalism) OR (hypocortisolism)] AND [(stiff person syndrome) OR (stiff man syndrome)]. The last literature search was performed on 12 July 2025, with restriction to English-language publications. References of relevant articles were also screened to identify additional reports. Inclusion criteria comprised eligible cases with (i) initial misdiagnosis of SPS and (ii) subsequent confirmation of hypoadrenalism. Exclusion criteria comprised studies (i) other than case reports, (ii) lacking full-text access, or (iii) with insufficient clinical details for data extraction. The screening of titles, abstracts, and full texts was performed by a single investigator (M.Z.).

### 2.3 Data collection

From each eligible case, data were collected on patient demographics (age, gender, and ethnicity), underlying etiologies, clinical manifestations, laboratory evaluation, imaging findings, EMG features, diagnoses, treatment, and prognosis. The clinical manifestations included musculoskeletal symptoms, their duration and distribution, and response to standard SPS management. Laboratory evaluation focused on plasma sodium level, endocrine panels, and anti-GAD or anti-amphiphysin antibodies detection. Imaging and EMG findings recorded the pituitary region MRI/CT (computed tomography) and motor-unit activity presentations, respectively. Treatment and prognosis data retrieved information on corticosteroid prescription, additional remedies, and therapeutic response.

## 3 Results

### 3.1 Demography and etiologies

A total of six previously reported cases of SPS mimic associated with endocrine dysfunction were identified (**Table 1**) (8–13). Together with our case, seven patients were included in the analysis. The overall median age was 61 years old (range, 42–68), with a median age of 66 years (range, 61–68) in men and 51 years (range, 42–51) in women. Sex distribution was relatively balanced, with a male-to-female ratio of 4:3.

**Table 1:** Characteristics and management of reported SPS mimic associated with hypopituitarism.

Author (year)	Age/sex/ethnicity	Clinical manifestations	Laboratory evaluation	Imaging findings	EMG findings	Diagnoses	Treatment	Prognosis
George et al. (1984) (8)	42/F English	Painful spasms, muscular rigidity, and flexion contracture over trunk and limbs for 3 years	Sodium 133 mmol/L↓ Marginally low T4 and T3 levels ACTH, GH, and PRL↓	Empty sella	Motor-unit discharge persisted during attempted antagonistic muscle contraction	Hypopituitarism secondary to a possible intrapartum pituitary necrosis	Transient improvement with baclofen Hydrocortisone 20 mg/day	Improvement of painful cramps and stiffness within 1 month
Chroni et al. (2000) (9)	51/F Greek	Muscle stiffness in lower trunk, proximal leg with stimulus induced painful spasms for 16 months	Insufficient response of TSH, FSH/LH, ACTH, and GH to stimulation with TRH, LHRH, CRH, and GHRH, respectively	Narrow sella turcica, atrophic and displaced pituitary gland	Bilaterally continuous and volitionally insuppressible firing of normal motor-unit potentials	Multiple anterior pituitary hormone deficiency probably due to postpartum pituitary necrosis	Partial benefit of standard SPS treatment Hydrocortisone 30 mg/day Thyroxine 100 μg/day	Complete disappearance of symptoms and almost normal EMG within 6 months
Odagaki et al. (2003) (10)	51/F Japanese	Flexion contracture of the legs and muscle stiffness in lumbar and thigh for 3 years	TSH 20 μIU/mL↑, FT4 0.6 ng/dL↓ No response of ACTH or cortisol to stimulation with CRH or insulin Negative anti-GAD antibody	No remarkable findings	Combined neuropathic and myopathic changes	Isolated ACTH deficiency Primary hypothyroidism	Prednisolone 5 mg/day	Rapid disappearance of musculoskeletal manifestation in 2 weeks
Lee et al. (2004) (11)	64/M Chinese	Muscle stiffness and painful spasms over lower back and legs precipitated by motion and tactile stimuli for 3 weeks	Morning cortisol, thyroxine, and anterior pituitary hormones↓ PRL↑	18 mm enhancing hypothalamic tumor with optic chiasm involvement	NA	Primary hypothalamic lymphoma Panhypopituitarism	Hydrocortisone 100 mg q8h Thyroxine 25 μg/day Surgery and radiotherapy	Improvement of pain and stiffness within 2 days
Kinoshita et al. (2010) (12)	61/M Japanese	Progressive tautness, muscle pain and arthralgia, flexion contractures of the lower limbs for 1 month	Sodium 129 mmol/L↓ ACTH 7.0 pg/mL↓, cortisol 0.5 μg/dL↓ No and low response in CRH and rapid ACTH test, respectively GH, PRL, LH, and FSH↑	Empty sella	Normal	Isolated ACTH deficiency.	Hydrocortisone 30 mg/day, gradually reduced to 15 mg/day	Improvement of musculoskeletal symptoms within a day
Goh et al. (2022) (13)	68/M Malaysian	Lower limb stiffness and truncal instability for 2 weeks	Sodium 122 mmol/L↓ Morning cortisol 24 nmol/L↓, inadequate response of ACTH to simulation tests FT4, FSH, LH, and testosterone↓ PRL 546 μU/mL↑	Multilobulated sellar lesion with a small hyperintensity area	NA	Pituitary macroadenoma Panhypopituitarism	Hydrocortisone 50 mg tid iv for 24 h → 20 mg tid po for 2 weeks → 15 mg/day Testosterone enanthate 250 mg Levothyroxine 25 and 50 μg/day	Improvement of stiffness and instability within 1 week
Present case	68/M Chinese	Rigidity of lower limbs and trunk with painful spasms precipitated by sensory stimuli for 6 months	Sodium 121 mmol/L↓ Low gonadal, thyroid, and adrenal cortex hormones PRL >204 μU/mL↑ Negative anti-GAD or anti-amphiphysin antibodies	Homogeneously enhanced pituitary with an empty sella	Spontaneous but transient self-relieved motor-unit potential firing	Prolactinoma Anterior hypopituitarism	No response to clonazepam IVIg 10 g/day and 25 g/day for 3 days each Hydrocortisone 200 mg/day for 5 days → methylprednisolone 1 g/day for 3 days and daily dose halving → prednisone 60 mg/day Levothyroxine 25 μg/day Bromocriptine 2.5 mg/day Surgery	Improvement of clinical manifestations within 2 days

ACTH, adrenocorticotropic hormone; CRH, corticotropin-releasing hormone; EMG, electromyography; F, female; FSH, follicle-stimulating hormone; FT3, free triiodothyronine; FT4, free thyroxine; GAD, glutamic acid decarboxylase; GH, growth hormone; GHRH, growth hormone-releasing hormone; IVIg, intravenous immunoglobulin; LH, luteinizing hormone; LHRH, luteinizing-hormone releasing hormone; M, male; NA, not applicable; PRL, prolactin; SPS, stiff-person syndrome; TRH, thyrotropin-releasing hormone; TSH, thyroid-stimulating hormone; T3, triiodothyronine; T4, thyroxine.

With respect to underlying etiologies, all male patients (*n* = 4) were diagnosed with hypopituitarism associated with various sellar masses, comprising both benign and aggressive lesions (11–13). The majority of female patients (*n* = 2) underwent postpartum hemorrhage and developed hypopituitarism secondary to Sheehan’s syndrome, except one diagnosed with isolated ACTH deficiency and primary hypothyroidism (8–10).

### 3.2 Clinical manifestations

Across cases, the most common musculoskeletal features included muscle stiffness and painful spasms, frequently involving the lower limbs and trunk. Precipitations, such as motion and tactile stimuli, were recorded in three of the cases. The duration of symptoms ranged from 2 weeks to 3 years prior to diagnosis. Laboratory data consistently showed endocrinological dysfunction, namely, the disruption of hypothalamic–pituitary–adrenal (*n* = 7), hypothalamic–pituitary–thyroid (*n* = 6), and hypothalamic–pituitary–gonad (*n* = 4) axes. Hyponatremia in four cases was accompanied by ACTH and/or cortisol deficiency. Negative serum anti-GAD (*n* = 2) or anti-amphiphysin (*n* = 1) antibodies were found in all available cases. Neuroimaging findings often revealed abnormalities in pituitary (*n* = 5) or hypothalamus (*n* = 1) regions, and EMG examinations demonstrated abnormal motor-unit activity (*n* = 3).

### 3.3 Therapeutic management

In the three cases that initially applied standard SPS treatment, including sedatives and anti-spasticity drugs, only transient or partial improvement if any response was observed. Subsequent treatment strategies primarily focused on hormone replacement therapy. Hydrocortisone was initiated, either orally or intravenously, at daily doses ranging from 10 to 300 mg in most patients (*n* = 6). Moreover, prednisolone 5 mg daily was prescribed alternatively in one case as well. In acute adrenal crisis, high-dose intravenous methylprednisolone and immunoglobulin were administered in combination. Concomitant hypothyroidism and hypogonadism were managed with thyroxine (*n* = 4) and testosterone (*n* = 1) replacements, respectively, and surgery or radiotherapy was applied in two patients with pituitary tumors. All the treatment received favorable outcomes, with rapid improvements of neuromuscular symptoms within days or weeks.

## 4 Discussion

### 4.1 Summary of clinical findings

We reported a patient with SPS mimic who initially experienced chronic progressive stiffness in the lower limbs and trunk, preceding an acute pituitary crisis characterized by severe hyponatremia and likely precipitated by subclinical infection. Combined with the unusual feature for classic SPS (e.g., oculomotor palsy), the clinical picture ultimately redirected the diagnosis to a movement disorder associated with hypopituitarism secondary to a pathologically confirmed prolactinoma.

Together with six published cases, the pooled findings delineate a distinctive subgroup of SPS mimics associated with endocrine dysfunction. The patients were typically middle-aged to elderly, with sex-specific etiologies. The pattern of sellar masses in male patients and obstetric complications in female patients appeared to underlie the approximately decade-earlier onset observed in females. Despite the phenotypic resemblance to SPS, the movement disorders secondary to hypoadrenalism displayed a distinctive triad: ACTH and/or cortisol deficiency (or related hyponatremia), negative anti-GAD (or anti-amphiphysin) antibodies, and limited response to benzodiazepine. These findings underscore the importance of scrutinizing pituitary function and morphology in atypical SPS cases, which may provide critical clues for hypoadrenalism as an alternative and treatable diagnosis.

### 4.2 Proposed pathophysiological mechanisms

#### 4.2.1 Macroprolactinoma with pituitary apoplexy

The pituitary neuroendocrine tumor, particularly macroadenoma, was regarded as a risk factor for pituitary apoplexy (14, 15). We speculated that our patient’s disease commenced with a macroprolactinoma undergoing asymptomatic partial tumor apoplexy before admission (16, 17). While the remaining non-infracted tumor continued to grow and protrude into the cavernous sinus, the necrotic portion was gradually resorbed and incurred an arachnoid herniation into the sella turcica. The secondary empty sella induced both compression on the pituitary and subclinical hormonal dysfunction (18, 19). Upon a virus infection, the sustained homeostasis decompensated under inflammatory stress, culminating in adrenal insufficiency.

#### 4.2.2 Glucocorticoid deficiency

Glucocorticoid deficiency has been hypothesized to directly impair the activity of membrane Na-K-ATPase, as well as diminish its permissive effect on the sensitivity of β-adrenergic stimulation on enhancing the Na-K pump function (20, 21). The restricted potassium influx favors depolarization and neuromuscular excitability, ultimately leading to stiffness and spasms. Another hypothesis suggests that glucocorticoids are indispensable in maintaining muscle properties and functions. Evidence from patients with Addison’s disease on conventional steroid treatment supported altered contractile properties and decreased endurance (22), and animal experiments further observed atrophy of type II fibers and slowed contractile properties following steroid treatment (23, 24). Together, adrenal insufficiency may contribute to muscle stiffness through disruptions in both energy metabolism and muscle properties. The insidious onset of contractures, predilection of leg and knees, and fixation of flexion position support this pathophysiological mechanism that the larger bulk of muscles are more vulnerable to such chronic metabolic disturbances.

The key to hypoadrenalism-related SPS mimic treatment focuses on hormone replacement therapy. Both first-choice hydrocortisone and alternative prednisone are highly prescribed, because of their short to medium effect as analogs of endogenous cortisol. The routine doses range from hydrocortisone 15 to 25 mg daily, depending on the disease course and severity. Emergency of adrenal crisis like our present case, however, demands immediate intravenous or intramuscular hydrocortisone for resuscitation regardless of overdose. Meanwhile, at least 40 mg of oral hydrocortisone daily should be additionally maintained until the underlying cause is resolved and the clinical condition stabilizes (25, 26).

#### 4.2.3 Hypothyroidism and Hoffmann’s syndrome

A complex form of long-standing hypothyroidism and hypothyroid myopathy, known as Hoffmann’s syndrome, may have also contributed to musculoskeletal symptoms (27, 28). The hallmark manifestations include proximal muscle weakness, stiffness, and pseudohypertrophy. Investigations typically demonstrate elevated creatine kinase levels and myopathic-featured EMG findings (29, 30). The hypothyroidism was confirmed in our case at the onset of discomfort, as well in other reported cases. In hypothyroidism management, it is crucial that glucocorticoid supplementation is initiated prior to thyroxine replacement, in case of precipitating a potential Addisonian crisis (31). Given that hypometabolism in hypothyroidism reduces both cortisol clearance and demand, imprudent thyroxine replacement can acutely deplete the availability of circulating cortisol while simultaneously increasing the requirement for it. In the context of concurrent adrenal insufficiency, relative adrenal insufficiency can further deteriorate into a life-threatening crisis. This emphasizes that the sequence of hormone replacement matters in such cases with multiple anterior pituitary hormone deficiencies.

### 4.3 Limitations

This study has several limitations. First, the pre-cortisol level from initial presentation was absent in our case, yet the temporal correlation indirectly supported our hypothesis. Given that the low-dose cortisol was administered prior to plasma hormone determination, it is likely that endogenous cortisol level was further lower than measured. Second, some included cases dated to distant years when clinical evaluations were limited, and the selected databases were unable to ensure exhaustive case retrieval. Nevertheless, the majority of the included studies provide solid clinical data supporting such a rare presentation. Future cohort and experimental studies are warranted to clarify the prevalence of endocrine-related SPS mimics, optimize diagnostic and management strategy, and elucidate the mechanisms by which hormonal deficiencies modulate neuromuscular excitability.

## 5 Conclusions

In conclusion, we presented a rare case in which SPS-like symptoms were the initial presentation of an underlying macroprolactinoma with hypopituitarism. To the best of our knowledge, this is the first published description of SPS mimic precipitated by a concurrent pituitary and adrenal crisis. The clinical course emphasizes the need for careful endocrine evaluation in atypical SPS presentations with abnormal electrolyte levels and pituitary imaging, while also highlighting the potential importance of prompt cortisol replacement in preventing life-threatening complications. Future directions should include epidemiological, clinical, and mechanistic studies, with this case illustrating the rarity, diagnostic challenges, and possible pathophysiology of endocrine-related SPS mimics.

## Data Availability

The original contributions presented in the study are included in the article/**Supplementary Material**. Further inquiries can be directed to the corresponding author.

## References

[1] Dalakas MC. Stiff-person syndrome and related disorders - diagnosis, mechanisms and therapies. Nat Rev Neurol. (2024) 20:587–601. doi: 10.1038/s41582-024-01012-3, PMID: 39227464

[2] Li Y Thakore N. An appraisal of electrodiagnostic studies in stiff person syndrome. J Clin Neuromuscul Dis. (2020) 22:84–9. doi: 10.1097/CND.0000000000000302, PMID: 33214393

[3] Jing X-Z Zhu D Zhang Y-Q Dong M Demyelinating Disease Study Group. Teaching video NeuroImages: electromyographic variation in stiff-person syndrome. Neurology. (2018) 90:e262. doi: 10.1212/WNL.0000000000004831, PMID: 29335314

[4] Baizabal-Carvallo JF Jankovic J. Stiff-person syndrome: insights into a complex autoimmune disorder. J Neurol Neurosurg Psychiatry. (2015) 86:840–8. doi: 10.1136/jnnp-2014-309201, PMID: 25511790

[5] Dalakas MC. Stiff person syndrome and GAD antibody-spectrum disorders. Contin (Minneap Minn). (2024) 30:1110–35. doi: 10.1212/CON.0000000000001457, PMID: 39088290

[6] Iglesias P. An update on advances in hypopituitarism: etiology, diagnosis, and current management. J Clin Med. (2024) 13:6161. doi: 10.3390/jcm13206161, PMID: 39458112 PMC11508259

[7] Fleseriu M Christ-Crain M Langlois F Gadelha M Melmed S. Hypopituitarism. Lancet (Lond Engl). (2024) 403:2632–48. doi: 10.1016/S0140-6736(24)00342-8, PMID: 38735295

[8] George TM Burke JM Sobotka PA Greenberg HS Vinik AI. Resolution of stiff-man syndrome with cortisol replacement in a patient with deficiencies of ACTH, growth hormone, and prolactin. N Engl J Med. (1984) 310:1511–3. doi: 10.1056/NEJM198406073102306, PMID: 6325914

[9] Chroni E Papadimitriou A Avramidis T Terentiou AE Tzioras C Divari R. Stiff-person like syndrome in a patient with multiple pituitary hormone deficiencies. Acta Neurol Scand. (2000) 102:403–5. doi: 10.1034/j.1600-0404.2000.102006403.x, PMID: 11125758

[10] Odagaki T Noguchi Y Fukui T. Flexion contractures of the legs as the initial manifestation of adrenocortical insufficiency. Intern Med (Tokyo Jpn). (2003) 42:710–3. doi: 10.2169/internalmedicine.42.710, PMID: 12924497

[11] Lee M-T Lee T-I Won JG-S Chau W-K Yang H-J Li J-C. Primary hypothalamic lymphoma with panhypopituitarism presenting as stiff-man syndrome. Am J Med Sci. (2004) 328:124–8. doi: 10.1097/00000441-200408000-00010, PMID: 15311173

[12] Kinoshita H Mizutani S Sei K Shimizu M Yasuda M Ohkubo T. Musculoskeletal symptoms and neurological investigations in adrenocortical insufficiency: a case report and literature review. J Musculoskelet Neuronal Interact. (2010) 10:281–5., PMID: 21116065

[13] Goh KG Yusof Khan AHK Nasruddin A. Stiff person-like syndrome: an unusual presentation of pituitary macroadenoma with panhypopituitarism. Case Rep Neurol. (2022) 14:157–61. doi: 10.1159/000522253, PMID: 35530378 PMC9035960

[14] Biagetti B Simò R. Pituitary apoplexy: risk factors and underlying molecular mechanisms. Int J Mol Sci. (2022) 23:8721. doi: 10.3390/ijms23158721, PMID: 35955859 PMC9369054

[15] Iglesias P. Pituitary apoplexy: an updated review. J Clin Med. (2024) 13:2508. doi: 10.3390/jcm13092508, PMID: 38731037 PMC11084238

[16] Kajal S Ahmad YES Halawi A Gol MAK Ashley W. Pituitary apoplexy: a systematic review of non-gestational risk factors. Pituitary. (2024) 27:320–34. doi: 10.1007/s11102-024-01412-0, PMID: 38935252

[17] Kinoshita Y Tominaga A Usui S Arita K Sugiyama K Kurisu K. Impact of subclinical haemorrhage on the pituitary gland in patients with pituitary adenomas. Clin Endocrinol. (2014) 80:720–5. doi: 10.1111/cen.12349, PMID: 24125536

[18] Padovano Sorrentino F Chiloiro S Giampietro A Bianchi A Pontecorvi A De Marinis L. Empty sella syndrome: an update. Pituitary. (2024) 28:13. doi: 10.1007/s11102-024-01475-z, PMID: 39738761

[19] Lundholm MD Yogi-Morren D. A comprehensive review of empty sella and empty sella syndrome. Endocr Pract. (2024) 30:497–502. doi: 10.1016/j.eprac.2024.03.004, PMID: 38484938

[20] Harbuz V Bihan H Salama J Reach G Cohen R. Flexion contractures possibly reflect the existence of hypocortisolism: two case reports. J Neurol. (2010) 257:1129–33. doi: 10.1007/s00415-010-5477-8, PMID: 20157722

[21] Kuo T McQueen A Chen T-C Wang J-C. Regulation of glucose homeostasis by glucocorticoids. Adv Exp Med Biol. (2015) 872:99–126. doi: 10.1007/978-1-4939-2895-8_5, PMID: 26215992 PMC6185996

[22] Jakobi JM Killinger DW Wolfe BM Mahon JL Rice CL. Quadriceps muscle function and fatigue in women with addison’s disease. Muscle Nerve. (2001) 24:1040–9. doi: 10.1002/mus.1108, PMID: 11439379

[23] Gardiner PF Botterman BR Eldred E Simpson DR Edgerton VR. Metabolic and contractile changes in fast and slow muscles of the cat after glucocorticoid-induced atrophy. Exp Neurol. (1978) 62:241–55. doi: 10.1016/0014-4886(78)90054-7, PMID: 153233

[24] Wilcox PG Hards JM Bockhold K Bressler B Pardy RL. Pathologic changes and contractile properties of the diaphragm in corticosteroid myopathy in hamsters: comparison to peripheral muscle. Am J Respir Cell Mol Biol. (1989) 1:191–9. doi: 10.1165/ajrcmb/1.3.191, PMID: 2624759

[25] Ftouh S Zucker M Tran A Tollerfield S Williams K Simpson H. Adrenal insufficiency: identification and management-summary of new NICE guidance. BMJ (Clin Res ed). (2025) 389:r330. doi: 10.1136/bmj.r330, PMID: 40312056

[26] Ebrahimi F Andereggen L Christ ER. Morbidities and mortality among hospitalized patients with hypopituitarism: Prevalence, causes and management. Rev Endocr Metab Disord. (2024) 25:599–608. doi: 10.1007/s11154-024-09888-8, PMID: 38802643 PMC11162375

[27] Sindoni A Rodolico C Pappalardo MA Portaro S Benvenga S. Hypothyroid myopathy: A peculiar clinical presentation of thyroid failure. Review of the literature. Rev Endocr Metab Disord. (2016) 17:499–519. doi: 10.1007/s11154-016-9357-0, PMID: 27154040

[28] Hs K Cheemalapati S Cr V. Hoffmann’s syndrome in subclinical hypothyroidism. J R Coll Phys Edinb. (2024) 54:26–8. doi: 10.1177/14782715231218033, PMID: 38078406

[29] Nalini A Govindaraju C Kalra P Kadukar P. Hoffmann’s syndrome with unusually long duration: Report on clinical, laboratory and muscle imaging findings in two cases. Ann Indian Acad Neur. (2014) 17:217–21. doi: 10.4103/0972-2327.132643, PMID: 25024579 PMC4090854

[30] Lee KW Kim SH Kim KJ Kim SH Kim HY Kim BJ. A rare manifestation of hypothyroid myopathy: hoffmann’s syndrome. Endocrinol Metab (Seoul Korea). (2015) 30:626–30. doi: 10.3803/EnM.2015.30.4.626, PMID: 26394732 PMC4722421

[31] Fountas A Andrikoula M Tsatsoulis A. A 45 year old patient with headache, fever, and hyponatraemia. BMJ. (2015) 350:h962–2. doi: 10.1136/bmj.h962, PMID: 25711278

