# Supplementary material for: Stiff-person syndrome mimic secondary to hypopituitarism: a case report and literature review
Source: Front Endocrinol (Lausanne). 2025 Oct 10;16:1664695. doi: 10.3389/fendo.2025.1664695 (PMC12549290; doi:10.3389/fendo.2025.1664695)
Supplement: Supplementary file 1 [file Table1.docx]

Supplementary Material

**SUPPLEMENTARY TABLE**

Plasma sodium and hormone levels before and two days after hydrocortisone treatment

|  | | Before (2024-01-27 to 2024-01-29) | After (2024-01-31) | Reference value |
| --- | --- | --- | --- | --- |
| Sodium level (mmol/L) | | 117.9-124.0 | 140.0 | 135-145 |
| Growth Hormone (ng/ml) | | - | 0.03 | 0.003-0.971 |
| Gonadal function | FSH (mIU/ml) | 0.97↓ | 1.19↓ | 1.27-19.26 |
|  | LH (mIU/ml) | 0.38↓ | 0.10↓ | 1.24-8.62 |
|  | PRL (ng/ml) | >204↑ | >204↑ | 2.64-13.13 |
|  | Estradiol (pg/ml) | 0.00 | 5.76 | <47 |
|  | Progesterone (ng/ml) | 0.03↓ | 0.00↓ | 0.14-2.06 |
|  | Testosterone (ng/dl) | 0.00↓ | 0.00↓ | 175-781 |
| Thyroid function | TSH (μIU/ml) | 4.07 | 4.61 | 0.55-4.78 |
|  | T4 (μg/dl) | 2.50↓ | 3.10↓ | 4.5-10.9 |
|  | FT4 (ng/dl) | 0.39↓ | 0.35↓ | 0.89-1.76 |
|  | T3 (ng/ml) | 0.85 | 0.48↓ | 0.6-1.81 |
|  | FT3 (pg/ml) | 2.55 | 1.49↓ | 2.3-4.2 |
| Adrenal function | ACTH (8am) (pg/ml) | - | 26.23 | 7.0-65 |
|  | Cortisol (8am) (μg/dl) | - | 5.97↓ | 6.7-22.6 |

ACTH, adrenocorticotropic hormone; FSH, follicle-stimulating hormone; FT3, free triiodothyronine; FT4, free thyroxine; LH, luteinizing hormone; PRL, prolactin; TSH, thyroid-stimulating hormone; T3, triiodothyronine; T4, thyroxine.
